# Bionomics of *Anopheles gambiae* complex (Diptera: Culicidae) and malaria transmission pattern in a pre-elimination area in South–Western Senegal

**DOI:** 10.1186/s12936-025-05411-9

**Published:** 2025-07-04

**Authors:** Moussa Diop, Youssouph Coulibaly, Omar Thiaw, Abdoulaye Kane Dia, Yaya Ibrahim Coulibaly, Modibo Sangaré, Ndèye Aita Ndoye, Moussa Diallo, Mame Fatou Tall, Mouhamadou Bassir Faye, Oumar Ciss, Seynabou Mocote Diédhiou, Ousmane Faye, Abdoulaye Diop, Abdoulaye Konaté, Badara Samb, Abdoulaye Niang, Lassana Konaté, Roger Clément Kouly Tine, El Hadji Amadou Niang

**Affiliations:** 1 Laboratoire d’Ecologie Vectorielle et Parasitaire, Département de Biologie Animale, Faculté des Sciences et Techniques, Université Cheikh Anta Diop de Dakar, BP 5005, Dakar, Sénégal; 2 Mali International Center for Excellence in Research (ICER), University of Sciences, Techniques, and Technologies of Bamako (USTTB), Bamako, Mali; 3 Service de Parasitologie et Mycologie, Faculté de Médecine, Pharmacie et Odontostomatologie (FMPOS), Université Cheikh Anta Diop de Dakar, Dakar, Sénégal

**Keywords:** *Anopheles gambiae*, Bionomics, Malaria, Mlomp, Pre-elimination, Transmission

## Abstract

**Background:**

Malaria remains a public health problem in many African countries. In Senegal, the Southern region had the highest malaria incidence and malaria-related deaths. The relationship between vector density and malaria transmission remains poorly understood in some specific areas. The aim of this study was to characterize the current entomological and transmission parameters with special emphasis on the *Anopheles gambiae* complex in the malaria pre-elimination area of Mlomp south-western Senegal.

**Methods:**

The study was conducted from July 2020 to February 2021 in Djicomol and Cadjinolle in the commune of Mlomp region of Ziguinchor, Senegal. Sampling was carried out using Human Landing Catches (HLC) and Pyrethrum Spray Catches (PSC). Ovaries were dissected to determine female parity rate. Infection status, blood meal sources and species molecular identification were determined using Enzyme-Linked Immunosorbent Assay (ELISA) and Polymerase Chain Reaction (PCR) techniques respectively.

**Results:**

A total of 6956 mosquitoes of the *An. gambiae* complex were collected, of which 6739 were by HLC (96.88%) and 217 by PSC (3.12%). The mean human biting rate was 36.98 bites/person/night (b/p/n) indoors and 43.25 b/p/n outdoors. Female biting activity was more frequent during the second half of the night. Mean parity rates were 24.83% indoors and 18.94% outdoors. The human blood index was estimated at 76.09%. Overall, *An. gambiae sensu stricto* (*s.s.*) was the most common species (75.08%). No female *An. gambiae* was found to be infected with *Plasmodium falciparum* in the sub-sample tested, thus no malaria transmission was recorded in Mlomp during the study period.

**Conclusions:**

The results alert malaria control programme to develop additional strategies for controlling these vectors, which show exophagic behaviours to effectively combat malaria.

## Background

Malaria remains one of the major public health problems globally, with an estimated 247 million cases in 2021, causing 619,000 deaths worldwide. The sub-Saharan African region remains the most affected region, with 234 million cases and 593,000 deaths [1, 2]. In 2022, a total of 358,033 malaria cases and 273 malaria-related deaths were reported in Senegal. Malaria incidence shifted from 2.67% to 3.12% between 2020 and 2021. However, between 2021 and 2022 it decreased from 3.12% to 2.02% [3, 4]. Malaria incidence was higher in the Southern part of the country, where 65% of cases and 30% of deaths are recorded [4].

Malaria transmission is especially modulated by environmental conditions and compatibility between vectors and parasites. In several areas in Senegal, various ecological situations are usually correlated with mosquito densities, particularly malaria vectors. The Sudanian zone is endowed with notable bioclimatic characteristics resulting in a proliferation of mosquito vectors in the rainy season [5, 6]. In certain areas, the presence of waterways, mangroves swamps and rice fields can locally influence malaria transmission patterns [7]. Species of the *Anopheles gambiae* complex are among the most efficient malaria vectors and are mainly responsible for malaria transmission in Senegal [8].

In the early 90 s, entomological surveys carried out in the south-eastern part of the country reported high entomological inoculation rates in Mlomp, with about 30 infective bites per person per year (Trape and Fontenille, pers. commun.). Nowadays, malaria burden has dropped sharply in this area, as a result of massive deployment of vector control measures, such as indoor residual spraying (IRS) and long-lasting insecticidal nets (LLINs) [4]. Malaria transmission is being significantly reduced and its elimination expected in this area. However, the scale-up of vector control interventions and other factors, such vegetation and animal presence, could lead to behavioural changes in malaria vector populations, hence the importance of updating entomological parameters of malaria transmission in all over the country, especially in Mlomp, a malaria pre-elimination setting, where epidemiological data were significantly lower compared with surrounded areas [4].

This study aimed to evaluate malaria entomological transmission in Mlomp, focusing on *An. gambiae sensu lato* (*s.l.*), in order to better guide decision-making for malaria elimination in this south-western part of Senegal.

## Methods

### Study sites

**Fig. 1:**
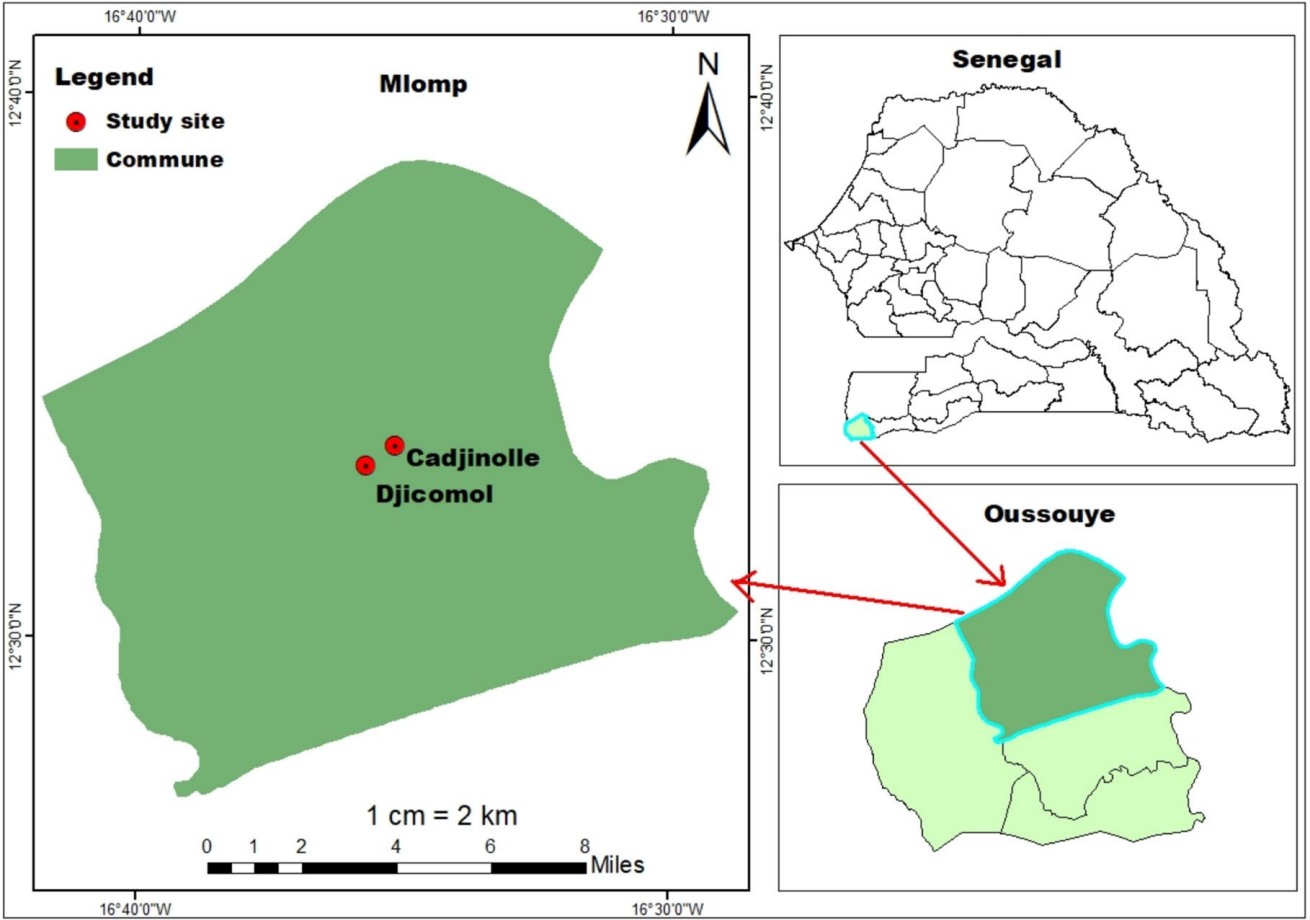
Localization of the study sites

The study was carried out in Mlomp, from July to December 2020 and February 2021, in neighborhoods of Djicomol and Cadjinolle (Fig. 1). Mlomp is a village in south-western Senegal, located in Oussouye (12°32 N, 16°37 W), one of the three departments of Ziguinchor region. Mlomp, which covers 70 square kilometres, lies 50 kms west of Ziguinchor city and 25 kms north of the border with Guinea-Bissau, around 500 kms from Dakar the capital city of Senegal [9]. The climate is Sudano-Guinean, with a rainy season from mid-June to November, and a dry season from November to mid-June. Annual rainfall is relatively high, varying between 1200 and 2000 mm. The temperatures vary between 15 and 18 °C in cool seasons, and between 27 and 32 °C in hot seasons [10].

In Mlomp, a large part of the inhabitants are farmers, with rice cultivation and market gardening as the main agricultural activities. These farming practices help to establish a particular ecosystem characterized by a microclimate favourable to the development of *An. gambiae*, with the creation of breeding sites in paddies and small water reservoirs in market gardening perimeters [10, 11]. During the rainy season, main breeding sites are puddles, hoofprints, permanent pools and water-filled potholes.

### Mosquito sampling and laboratory processing

Mosquito sampling was carried out in Djicomol and Cadjinolle sites, on monthly basis, using Human Landing Catches (HLC) and Pyrethrum Spray Catches (PSC) techniques. For HLC activities, three households were randomly selected as capture points where one room was used at each point. Capture sessions took place in two consecutive nights, between 8 p.m. and 6 a.m., simultaneously involving two capture operators one placed inside the room and the other outside. At each capture point, two capture operators worked in pairs between 8 p.m. and 1 a.m., before replaced by another pair working during the second half of the night, i.e. from 1 a.m. to 6 a.m. In order to minimize biases linked to the differential attractiveness of the operators, the latter were rotated by alternating pairs between the time slots (8 p.m./1 a.m. and 1 a.m./6 a.m.) and the capture points. A supervisor was appointed to ensure that collection activities were correctly conducted, by monitoring work instructions and offering coffee to the capture operators to keep them from dozing off. Collected mosquitoes were counted and identified using conventional keys [12]. A subset of host-seeking females (n = 1423) was dissected to determine the physiological age [13].

Indoor resting females were collected using PSC in ten randomly selected rooms both in Djicomol and Cadjinolle, from 7 a.m. to 9 a.m. The technique consisted of laying white sheets on the floor of selected rooms, then spraying inside with overactivated solution of pyrethroids (by adding piperonyl butoxide), after first taking out food and blocking all exits that could serve as an escape for mosquitoes. Ten minutes after spraying, the sheets were carefully removed and *Anopheles* mosquitoes were collected using flexible forceps and then placed into numbered Petri dishes. Collected *Anopheles* mosquitoes were identified and classified according to the repletion state (unfed, fed, half-gravid and gravid).

At the laboratory, specimens were separately dissected. Head-thoraces, fed female abdomens and legs or wings, were used to determine *Plasmodium* infection rates, feeding host and species identification, respectively. Blood meal origin of indoor resting females was determined by Enzyme-Linked Immunosorbent Assay (ELISA) method [14]. The infectivity of host-seeking females was detected using another ELISA technique that targeted circumsporozoïtique antigen (ELISA-CSP) as described by Wirtz et al*.* [15]. Genomic deoxyribonucleic acid (DNA) was extracted from individual mosquitoes using the protocol of Collins et al. [16]. The sibling species of *An. gambiae* complex were identified using polymerase chain reaction (PCR) as described by Wilkins et al*.* [17].

### Data analysis

Human-biting rates were calculated by dividing the number of *Anopheles* females caught on human by the number of mosquito collectors who operated per night. Indoor resting densities were obtained by dividing the number of females collected inside dwellings by the number of rooms sprayed. Infection rates were obtained by dividing the number of infected female (ELISA-CSP positive) by the number of tested females. Parity rates were calculated by dividing the number of parous females by the total dissected. Entomological inoculation rates were expressed as the product of infection rates and human-biting rates. Human blood index was obtained as the ratio between the number of blood meals taken from humans and the total number of blood meals identified. Data were entered into an Excel database. The Student’s test (t-test) was used to compare mean indoor and outdoor densities. The average number of parous females were also compared between indoor and outdoor biting mosquitoes using t-test. Monthly variations of abundance were compared using X-squared test. All statistical analysis were performed using R software (version 4.3.0). The significance level was set at 5%.

## Results

### Diversity of anopheline fauna and specific composition of *An. gambiae* complex

**Fig. 2:**
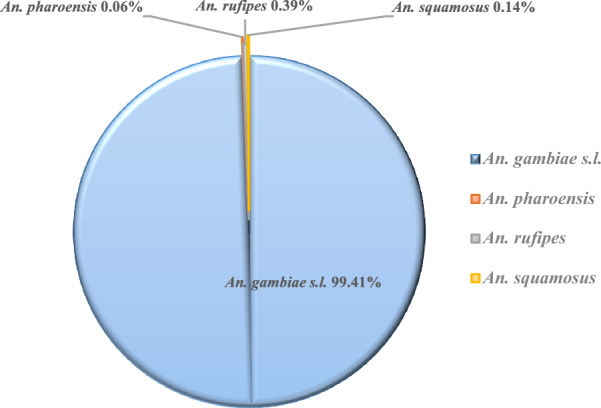
Diversity of collected *Anopheles* species

A total of 6997 *Anopheles* mosquitoes were collected. Species of the *An. gambiae* complex were predominant (99.41%, n = 6956), followed by *Anopheles rufipes* (0.39%, n = 27), *Anopheles squamosus* (0.14%, n = 10) and *Anopheles pharoensis* (0.06%, n = 4) (Fig. 2).

PCR performed on a sub-sample of 2087 specimens belonging to the *An. gambiae* complex revealed the presence of *Anopheles gambiae s.s.* (75.08%, n = 1567), *Anopheles arabiensis* (4.50%, n = 94), *Anopheles coluzzii* (2.06%, n = 43), *Anopheles melas* (1.34%, n = 28), and *An. gambiae/An. coluzzii* hybrids (17.01%, n = 355).

### Variation of entomological parameters and malaria transmission

#### Relative abundance

Out of 6956 females *An. gambiae* collected, 96.88% (n = 6739) were collected by HLC and 3.12% (n = 217) by PSC. Monthly variations of the abundance of *An. gambiae* were observed (X-squared = 9863, df = 6, *p* = 2.2e−16). In July, HLC collections showed low mosquito abundance both indoors (3.51%) and outdoors (3.17%). Mosquito abundance increased sharply in August and in September both indoors (30.68% and 43.59%) and outdoors (27.47% and 51.47%). In October the abundance decreased significantly to 21.76% indoors and 17.62% outdoors. In December and February, a few host-seeking mosquitoes were collected indoors (0.03%). For indoor resting females, the relative abundance was 4.15% in July, and reached 52.53% in August and gradually decreased to 0% in February (Table 1).

**Table 1:** Variation of relative abundance of *An. gambiae* complex

Month	HLC				PSC	
	Indoors		Outdoors			
	n	%	n	%	n	%
July 2020	109	03.51	115	03.17	09	04.15
August 2020	953	30.68	998	27.47	114	52.53
September 2020	1,354	43.59	1,870	51.47	56	25.81
October 2020	676	21.76	640	17.62	24	11.06
November 2020	10	00.32	10	00.27	09	04.15
December 2020	03	00.10	00	00.00	05	02.30
February 2021	01	00.03	00	00.00	00	00.00
Total	3106		3633		217	

n: number collected; %: percentage; HLC: human landing catches; PSC: pyrethrum spray catches

#### Human-biting rates

**Fig. 3:**
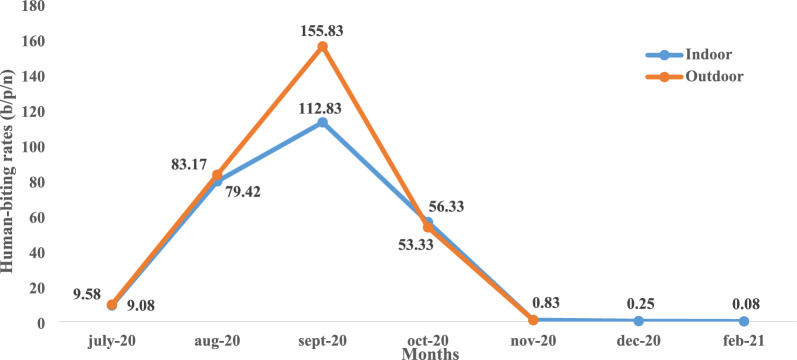
Variation of human-biting rates of members of the *An. gambiae* complex

**Fig. 4:**
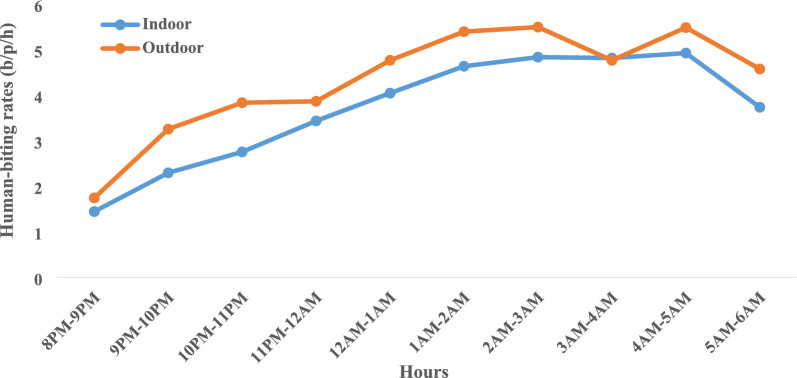
Variation in nocturnal activity of *An. gambiae* complex indoors and outdoors

A total of 168 person-nights were used, during 14 capture sessions, in each collection site (Djicomol and Cadjinolle). The mean human-biting rate (HBR) was 40.11 bites per person per night (b/p/n). Globally, there was no significant difference between indoor (36.98 b/p/n) and outdoor (43.25 b/p/n) HBRs (t = − 0.22, df = 11.31, *p* = 0.83), indicating opportunistic biting behaviour of *An. gambiae* females. However, monthly HBR variations were recorded. In July, mean HBR was 9.08 b/p/n indoors and 9.58 b/p/n outdoors, whereas it increased significantly in August both indoors (79.42 b/p/n) and outdoors (83.17 b/p/n). Two peaks were recorded in September (112.83 b/p/n indoors and 155.83 b/p/n outdoors). The HBR dropped to 56.33 b/p/n indoors and 53.33 b/p/n outdoors in October and reached 0.83 b/p/n both indoors and outdoors in November. It continued to fall steadily to 0.25 b/p/n in December up to 0.08 b/p/n indoors and 0 b/p/n outdoors in February (Fig. 3). Hourly variations of HBRs showed that *An. gambiae* were more aggressive during the second half of the night (1 a.m. to 6 a.m.), representing 60% of females captured (Fig. 4). The biting activity was higher outdoors excepted from 3 a.m. to 4 a.m. where indoor and outdoor HBRs were similar. Two peaks were noted outdoors, one between 2 a.m. and 3 a.m., and another between 4 a.m. and 5 a.m. However, a single peak was observed indoors between 4 a.m. and 5 a.m. The highest biting rate both indoors and outdoors was obtained during the second half of the night.

#### Parity rates

Globally, the mean parity rate was 21.92%. No significant difference (t = 0.25; df = 11.94, *p* = 0.802) in mosquito physiological age was recorded between indoors (24.83%) and outdoors (18.94%).

In July, the parity rate was 46.81% indoors and 48.84% outdoors and then dropped from August to September to 17.72% indoors and 0.1% outdoors. A slight increase in October (27.72%) and a decrease in November (11.11%) were noted indoors, while there was an increase outdoors (22.22%) in the same period (Table 2).

**Table 2:** Physiological ages of female *An. gambiae* complex indoors and outdoors

Month	Indoors				Outdoors			
	n	Dis	P	PR (%)	n	Dis	P	PR (%)
July 2020	109	47	22	46.81	115	43	21	48.84
August 2020	953	223	58	26.01	998	224	51	22.77
September 2020	1354	237	42	17.72	1870	240	24	00.10
October 2020	676	202	56	27.72	640	186	35	18.82
November 2020	10	09	01	11.11	10	09	02	22.22
December 2020	03	02	00	00.00	00	00	00	00.00
February 2021	01	01	00	00.00	00	00	00	00.00
Total	3106	721	179	24.83	3633	702	133	18.94

n: number collected; Dis: dissected; P: parous; PR: parity rate; %: percentage

#### Indoor resting densities

**Fig. 5:**
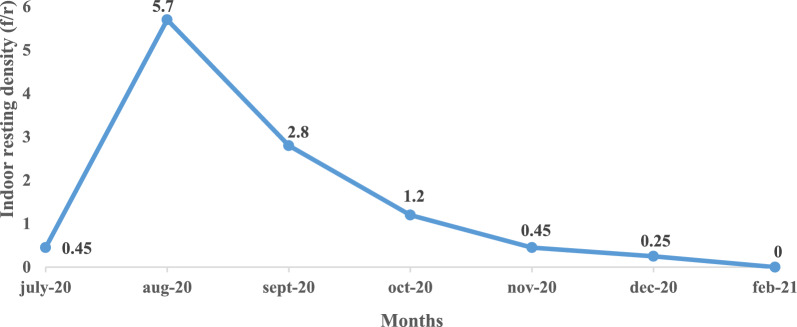
Resting density variation of *An. gambiae* complex population

The mean indoor resting density (IRD) was estimated at 1.55 female per room (f/r). This low IRD suggested that a large proportion of *An. gambiae* females rested outdoors (exophilic females). Monthly variations of IRDs were noted. In July, mean IRD was very low (0.45 f/r),

but it increased significantly in August (5.7 f/r), before decreasing. In February no indoor resting female was collected (Fig. 5).

#### Trophic preferences

Out of the 136 *An. gambiae* tested, 92 were identified, 67 feeding on humans, 16 on beef, two on chicken, one on sheep and three on both human and sheep (mixed meals). The human blood index was estimated at 76.09% (Table 3).

**Table 3:** Origin of blood meals for female *An. gambiae* complex

Tested (n)	Identified (n)	Negative (n)	Vertebrate hosts					Mixed	HBI (%)
			H	B	S	C	Ho	H/S	
136	92	47	67	16	1	2	0	3	76.09

n: number; H: human; B: beef; S: sheep; C: chicken; Ho: horse; H/S: human-sheep; HBI: human blood index; %: percentage

### Infectivity of *An*. *gambiae* and malaria transmission

Amongst 1894 *An. gambiae* specimens tested by ELISA-CSP, no positive was recorded. Thus, no female was found infected by *P. falciparum* and the entomological inoculation rate was zero in both collection sites (Djicomol and Cadjinolle).

## Discussion

This study showed that *An. gambiae s.s.* was the predominant species within *An. gambiae* complex in Mlomp. The abundance of this species was the highest during the rainy season. On the other hand, high hybridization rates between *An. gambiae* and *An. coluzzii* were recorded. *Anopheles gambiae* showed an outdoor biting behaviour and was more aggressive during the second half of the night. Its population consisted mainly of young females, with highest indoor resting densities recorded in August. *Anopheles gambiae* females were highly anthropophilic. However, no female was found infected with *P. falciparum*, and as a result, no malaria transmission was detected.

The predominance of *An. gambiae* in humid areas, has already been reported by several authors [4, 7, 12, 18, 19], most often associated, with bioclimatic conditions which are conducive to development of that species [8]. The high proportion of *An. gambiae*/*An. coluzzii* hybrids should indicate that the latter species are still under reproductive isolation process in this area. A similar percentage of hybrids (18.30%) has been noted by Caputo et al. [20] in Ivory Coast. Several studies have demonstrated this uncomplete reproductive isolation between these two species, with high hybridization rates [21, 22]. In other areas, reproductive isolation between *An. gambiae s.s.* and *An. coluzzii* was more extensive, with low hybrid frequencies [19, 23–27].

The highest species abundances (both HLC and PSC combined) were observed during the rainiest periods, as reported by previous studies [6, 8, 12, 28]. This high abundance can also be explained by the presence of rice fields, which provide suitable breeding sites and increase the availability of pre-imaginal stages. Irrigation of these fields during rainy season leads to an increase in vector population density [29].

The exophagic behaviour of *An. gambiae* can be explained by the fact that inhabitants stayed outside for a long time before going to bed. The use of mosquito nets in dwellings also favors exophagy. The same phenomenon has been observed in the Sine Saloum region by Niang et al. [30], in Ngari (Kedougou) by Dia et al*.* [31], in Ambohimena (Madagascar) by Rajaonarivelo et al*.* [32], in Ivory Coast by Assouho et al. [33]. This relative exophagy trend [4] of *An. gambiae* complex is in contrast to what Faye [34] has reported. Aggressive density has positively correlated with the abundance of *An. gambiae* females, the latter being regulated by the rainfall regime [30, 35].

The high level of aggressivity during the second half of the night can be explained by the availability of the human host at this time. This biting behaviour demonstrates the extent to which *An. gambiae* complex is dependent on human habits. These results corroborate the observations of Trape (pers. commun.), who has recorded more than 110 bites per person per hour in Mlomp during the second half of the night in 1989. Faye [34] has noted maximum aggressivity of *An. gambiae* females between 2 a.m. and 3 a.m. in the Bignona marigot valley. Robert et al*.* [35] have noted maximum aggressivity between 1 a.m. and 2 a.m. at Niakhar. Faye et al*.* [36] have noted two peaks of aggressivity, one between 1 a.m. and 2 a.m. and the other between 6 a.m. and 7 a.m. In Dielmo, Konate et al*.* [37] have observed a maximum aggressivity of *An. gambiae* during the second half of the night.

The low parity rate contrasts with the 66% reported by NMCP [4] in the Sudano-Guinean area, and with Konate et al*.* [37] have obtained an average parity rate of 72% in Dielmo. Faye et al. [38] have found parity rate of up to 73.8% in the Senegal River delta. The drop in the parity rate during period of heavy rain could be due to new emergence of females (neonate) at this time [29]. Monthly variations in parity rate and aggressive density of *An. gambiae* females were negatively correlated (low longevity coupled with high density). These observations are in clear agreement with the results of Faye [34] in the area. The same phenomenon has observed in Copargo in North**-**East Benin [39]. The parity rates showed that the *An. gambiae* complex population was made up mainly of neonate (young) females. In other words, few females had reached the epidemiologically relevant age, with a very low of transmitting malaria parasites.

The high trophic preference of the species for humans was consistent with the studies of Faye et al*.* [7] and Konate et al*.* [37] respectively in Wassadou and Dielmo. In the Sudanian and Sudano-Guinean areas of the country, human blood index of *An. gambiae s.l.* exceeding 80% have been noted [4, 8]. This high human blood index reflects significant human-vector contact, despite the use of mosquito nets. The high percentage of unidentified meals (34.56%) would belong to not targeted animal hosts such as dogs, pigs and donkeys also present in the area. This could explain the high zoophilic rates recorded in the study site. In addition, a very low level of IgG antibodies in the eluates could occur because of a probable digestion of blood meal in field-collected specimens.

The absence of infected females was consistent with the work of several authors (Carrara et al. [40] at Souhloul (Saint Louis), Manga et al*.* [41] at Nkol Mefou (Yaounde), Faye et al*.* [38] in the Senegal river delta, Faye et al*.* [7] at Thiaye in the Niayes, Diop et al*.* [42] in the Saloum delta, Rajaonarivelo et al. [32] at Ambohimena (Madagascar), Jawara et al*.* [43] in Gambia). The low longevity of the species (21.92% parity rate out of 1423 dissections) could partly explain this absence of infected females, as shown by Akogbeto [44] in Ganvié (Benin). The main explanation given for the absence of infected females is that Mlomp is located in a malaria pre-elimination area. Transmission has not been detected in Mlomp, but the question remains as to whether it exists or not. This was the case in Thiaye from September 1992 to October 1993 [7]. These results contradict those of Trape et al*.* [45], who have found 30 infected bites per person per year. Agnamey et al*.* [46] have found 25 infected bites per person per year, and that transmission lasted all year round, peaking during the rainy season. The NMCP [4] has reported 0.193 infected bites per person per night. This Sudanian area of the country has often characterized by high seasonal transmission, with entomological inoculation rates more than 100 infected bites per person per year [8].

The study did not detect any malaria transmission in Mlomp. It would be interesting to investigate infectivity of indoor resting females to maximize the change of finding infected specimens. Another limitation of the study is the fact that the study sites were in a malaria pre-elimination area. It would be interesting (i) to carry out a similar study in the south, in where malaria incidences are the highest in the country; and (ii) to study the exophilic population of *An. gambiae* complex, a major problem in the fight against malaria.

## Conclusions

These results provide information on the specific composition of the *An. gambiae* complex and the predominant species in the area. They also allow to determine the dynamics of *An. gambiae* complex and entomological parameters. Increasing of parameters such as aggressive density and human blood index are transmission risk factors, while decreasing of parity rate, circumsporozoitic index and the entomological inoculation rate are limiting malaria transmission. In Mlomp, it should be noted that high *Anopheles* densities do not necessarily result in increasing of malaria transmission. These results were consistent with the current epidemiological status of this area which is a malaria pre-elimination area, and will serve as a reference for future studies. The results also alert malaria control programme to develop additional strategies for controlling these vectors, which show exophagic behaviours, to effectively combat malaria.

## Data Availability

No datasets were generated or analysed during the current study.

## Abbreviations

b/p/n: Bite per person per night
CSP: Circumsporozoïtique surface protein
DNA: Deoxyribonucleic acid
ELISA: Enzyme-Linked Immunosorbent Assay
f/r: Female per room
HBR: Human-biting rate
HLC: Human landing catches
IgG: Immunoglobulin G
IRD: Indoor resting density
IRS: Indoor residual spraying
LLIN: Long-lasting insecticide-treated net
NMCP: National Malaria Control Programme
n: Number
PCR: Polymerase chain reaction
PSC: Pyrethrum spray catches
s.l.: Sensu lato
s.s.: Sensu stricto
WHO: World Health Organization
